# Sodium Intake and Hypertension

**DOI:** 10.3390/nu11091970

**Published:** 2019-08-21

**Authors:** Andrea Grillo, Lucia Salvi, Paolo Coruzzi, Paolo Salvi, Gianfranco Parati

**Affiliations:** 1 IRCCS Istituto Auxologico Italiano, Cardiology Unit, 20100 Milan, Italy; 2 IRCCS Policlinico San Matteo Foundation, University of Pavia, 27100 Pavia, Italy; 3 Department of Medicine and Surgery, University of Parma, 43121 Parma, Italy; 4 Chair of Cardiovascular Medicine, Department of Medicine and Surgery, University of Milano-Bicocca, 20100 Milan, Italy

**Keywords:** arterial stiffness, endothelial function, hypertension, salt-sensitivity, salt intake, sodium intake, sympathetic activity

## Abstract

The close relationship between hypertension and dietary sodium intake is widely recognized and supported by several studies. A reduction in dietary sodium not only decreases the blood pressure and the incidence of hypertension, but is also associated with a reduction in morbidity and mortality from cardiovascular diseases. Prolonged modest reduction in salt intake induces a relevant fall in blood pressure in both hypertensive and normotensive individuals, irrespective of sex and ethnic group, with larger falls in systolic blood pressure for larger reductions in dietary salt. The high sodium intake and the increase in blood pressure levels are related to water retention, increase in systemic peripheral resistance, alterations in the endothelial function, changes in the structure and function of large elastic arteries, modification in sympathetic activity, and in the autonomic neuronal modulation of the cardiovascular system. In this review, we have focused on the effects of sodium intake on vascular hemodynamics and their implication in the pathogenesis of hypertension.

## 1. Sodium Intake and Blood Pressure Values

Available evidence suggests a direct relationship between sodium intake and blood pressure (BP) values [1,2,3,4]. Excessive sodium consumption (defined by the World Health Organization as >5 g sodium per day [5]) has been shown to produce a significant increase in BP and has been linked with onset of hypertension and its cardiovascular complications [6,7]. Conversely, reduction in sodium intake not only decreases BP levels and hypertension incidence, but is also associated with a reduction in cardiovascular morbidity and mortality [8]. A large meta-analysis [9] showed that modest reduction in salt intake for four or more weeks causes a significant fall in BP in both hypertensive and normotensive individuals, irrespective of sex and ethnic group, and larger reductions in salt intake are linked to larger falls in systolic BP [9]. However, the current health policies have not reached an effective achievement for the reduction of dietary sodium in the population and the positive effects of a reduced sodium intake on BP levels tend to decrease with time, owing to poor dietary compliance. 

The pathophysiological link between sodium intake and increase in BP values has been widely debated. Increased salt consumption may provoke water retention, thus leading to a condition of high flow in arterial vessels. The mechanism of pressure natriuresis has been proposed as a physiologic phenomenon where an increase in BP in the renal arteries causes increased salt and water excretion [10]. This hemodynamic load, as studies with animal models have shown [11,12], may lead to an adverse microvascular remodeling by the effects of increased BP levels. High sodium intake and increased BP levels are linked by changes in vascular resistances, but the mechanisms controlling this phenomenon may not be only viewed as a reflex pressor response aimed at increasing sodium excretion. Excessive salt intake may induce several adverse effects, causing microvascular endothelial inflammation, anatomic remodeling, and functional abnormalities, even in normotensive subjects [13]. More recent studies have shown that changes in sodium plasma levels do not only exert their effects on small resistance arteries, but may also affect the function and structure of large elastic arteries. The issue of salt-sensitivity, which refers to individual susceptibility in terms of BP variations following changes in dietary salt intake, has also been recently debated in its pathophysiological background and clinical implications [14,15].

In this paper, we have reviewed the evidence regarding the effects of sodium intake on arterial function, and their implication in the pathogenesis of hypertension. We have first addressed the debate on salt-sensitivity, in light of recent evidence, and then discussed the effects of sodium handling on arterial function and structure.

## 2. Low Sodium Intake and Cardiovascular Risk

Over the years, the evidence of a close relationship between high sodium intake and hypertension, and high sodium intake and increased cardiovascular risk and mortality, has become increasingly consolidated. For this reason, we are used to consider that the lower the sodium intake is, the better the patient prognosis is. However, the studies that are beginning to shake the foundations of this historic fortress are growing in number. Actually, in the analysis of this topic, several cohort studies [16,17,18] and meta-analyses [19,20] have shown that the relationship between sodium intake and poor patient prognosis have not a linear trend, but rather describe a J-shape curve. In these studies, an increased risk not only in high sodium intake, but also in significantly low sodium intake levels is underlined. To reach this declaration, large patient populations have been studied, including various types of healthy patients or those with different co-morbidities (i.e., diabetes, vascular disease, hypertension population), with wide numbers in all subgroups. 

The relationship between cardiovascular events and sodium intake was derived from baseline urinary sodium excretion on a 24-h urine collection: urinary sodium excretion less than 3 g/day is considered to reflect a low sodium dietary intake. It was observed that a poor patient prognosis is associated with either a very high and a very low 24h urinary sodium excretion. This relationship does not depend on BP, aging, diabetes, chronic kidney disease, or cardiovascular disease. Mente et al. [19] reported that only patients with arterial hypertension have a high cardiovascular risk associated with high sodium intake, while this association was not confirmed in patients without hypertension [19].

Mechanisms linking high sodium intake and cardiovascular adverse events are well known; less defined are those that justify a relationship between low salt intake and high mortality. Sodium is an indispensable cation, essential to the action potential of all cells in the body, and its homoeostasis is under tight physiological regulation. Sodium intake is governed by neural mechanisms that regulate intake of sodium and related homoeostatic systems, and so although extreme reductions in sodium intake are possible in controlled settings for short periods, this is unlikely to be sustainable in everyday life in the long-term. Thus, as for all our body components, there may be an optimal range for its intake, below which the human body starts being damaged, at variance from what happens in case of intake of, or exposure to, potentially toxic external substances, such as tobacco smoke, drugs or environmental pollutants.

In experimental models, it is known that sodium restriction results in increased atherosclerosis [21]. In humans, the relationship between salt restriction and increased renin-angiotensin-aldosterone system activation has been described [22,23], as well as the relationship with increased sympathetic activity [24] and insulin resistance [25,26,27]. High renin concentrations and increased levels of catecholamines have been reported in studies in poor sodium intake population. On the other hand, several studies have shown that increases of renin, aldosterone, and catecholamines are all associated with increased cardiovascular disease events and mortality [24,28]. Regarding sympathetic activity, sodium intake restriction is associated with a persistent attenuation of the muscle sympathetic nerve activity responses to baroreceptor stimulation and deactivation [28].

Furthermore, there is a significant correlation between the reduction in baroreflex sensitivity and the increase of concomitant muscle sympathetic nerve activity. Accordingly, a reduced ability of this reflex to obtain a proper downregulation of sympathetic tone leads to the sympathostimulating effect of a very low sodium intake. As described in other studies evaluating high sodium intake, the increase in muscle sympathetic nerve traffic due to very low sodium intake is also associated with an increase in plasma norepinephrine, and a drastic reduction in sodium intake has been reported to cause, in man, an increase in renal norepinephrine removal. Moreover, sodium restriction causes insulin resistance; this may be the result of sympathetic activation but, in turn, increased insulin levels may themselves have a sympathoexcitatory influence. Lastly, low salt supply causes a reduction of central venous pressure, which may lead to an activation of the sympathetic system via unloading of cardiopulmonary receptors.

To sum up, predicting the net clinical effect of low sodium intake based on only considering the effects of sodium on BP might not provide a comprehensive view of its effects on cardiovascular disease and mortality, especially within the range of sodium intake that affects the renin system (<4 g/day). In other words, the effects of sodium intake level on clinical outcomes are only partly mediated through its effects on BP. For a full understanding of the clinical impact of sodium intake it is also necessary to consider other mechanisms that might be at play. In particular, while the potential harm of sodium excess may be BP-driven, the potential negative effects of a low sodium intake may be mediated by elevated renin-aldosterone activity and sympathetic neural activation.

Numerous methodological concerns have arisen from studies that have underlined the J-shape of the sodium intake and cardiovascular events’ relationship [29,30]. It has been remarked that a single morning urine sample may offer an inaccurate measure of usual sodium intake, ignoring day-to-day variability in sodium intake, diurnal variation in sodium excretion, and the effects of medications. Another confounder could come from the fact that other prognostically negative factors might be activated in the low sodium intake interval because of dietary advice or poor appetite, or in the high sodium intake interval because of concomitant high caloric intake (typical of overweight or diabetic patients), which could have contributed to increased mortality in the low- and the high-sodium groups respectively (reverse causality). However, most studies took measures to adjust for such confounding elements.

Recent findings further support the calls for caution before applying salt restriction universally. Although more studies have confirmed the benefit of reducing sodium intake in hypertensive subjects with a high salt intake, it is unclear whether the remaining more than 90% of the population will profit from dietary sodium reduction. Therefore, until new robust data emerge from large trials, it might be prudent to recommend reduction in sodium intake only in those with high sodium intake and with hypertension. In other words, it would perhaps be more correct to start discussing about "inappropriate" rather than "excessive" salt intake.

## 3. Hypertension and Salt-Sensitivity

Nearly half a century ago, Guyton and Coleman proposed that whenever arterial pressure is elevated, the pressure natriuresis mechanism enhances the excretion of sodium and water until blood volume is reduced adequately in order to return BP to normal values [31]. According to this premise, hypertension may occur only when the ability of the kidney of excreting sodium is impaired. Further evidence has shown that the BP response to changes in salt intake in diet has a significant variability among individuals in the general population. This phenomenon was defined as salt-sensitivity of BP. Strains of rats whose BP was either sensitive or resistant to changes in sodium intake were developed [32], thus establishing a genetic background for the phenomenon of salt-sensitivity. However, the BP response to changing salt intake display marked inter-individual variability [33,34], and thus salt-sensitivity behaves as a continuous parameter at a population level. Although the role of salt-sensitivity is of increasing interest both in research and in a clinical setting, the existing methods to identify salt-sensitivity and resistance may be imprecise and the definitions of “salt-sensitive” and “salt-resistant” hypertension are based on relative inaccurate approaches. Generally, the definition of salt-sensitivity is based on the BP response to moderate reduction and increase of salt intake. For the clinical evaluation of BP salt-sensitivity, a commonly used protocol in clinical research is the test of Grim and Weinberger [6], which has been the reference test for the last decades. According to this protocol, patients are prescribed to follow a diet with high sodium (200 mmol NaCl per day) and with a low sodium intake (30 mmol NaCl per day) diet, each for one week, with the quantification of 24-hour urinary sodium excretion on the last day of each diet week [35,36]. A modified protocol with a more rapid execution was proposed and tested, and was able to correctly predict a significant BP response to dietary salt restriction [37]. The prescription of a long-term reduction in sodium intake is often limited because of insufficient compliance of patients to the dietary instructions, and the follow-up may be challenging for both patients and physicians. This is the reason why, more recently, Castiglioni et al. proposed a protocol based on ambulatory BP monitoring (ABPM) for a simplified clinical screening of BP salt-sensitivity [38]. These authors hypothesized that in a population following a high-sodium diet, individuals with a marked salt-sensitivity may display an altered circadian profile, with a less pronounced nocturnal dipping, as a consequence of retention of sodium and water in the daytime, accompanied by an elevated mean 24-hour heart rate. By using such an “ambulatory salt-sensitivity index”, based on the combination of reduced nocturnal BP dipping and elevated 24 h heart rate, they established three classes of risk for salt-sensitivity (low, intermediate, and high) by combining the BP-dipping and heart rate levels observed during a 24h ABPM without need of changing the dietary sodium content. This index was validated through the observation that the prevalence of sodium-sensitive patients, evaluated with a traditional test, increased significantly from the class with the lowest risk for salt-sensitivity (25% of prevalence) to the intermediate risk (40%) and high risk (70%) classes, as defined by this index of ambulatory salt-sensitivity. Thus, by performing ABPM in conditions of usual daily life and with habitual diet, some useful information on the degree of salt-sensitivity of patients with hypertension may be available with an easy and direct method, without resorting to a traditional approach requiring a demanding salt-sensitivity test. 

In both normotensive and hypertensive persons, current evidence suggests that salt-sensitivity is associated with an increased cardiovascular risk. The risk for developing hypertension is higher in normotensive men with a more pronounced salt-sensitivity at baseline, in a long-term follow-up [39]. Moreover, in patients with essential hypertension, the prevalence of severe hypertensive target organ damage was higher among salt-sensitive patients [40]. Cardiovascular morbidity and mortality were found to be higher both in hypertensive and even in normotensive individuals with a higher degree of salt-sensitivity [34,41]. A cluster of possible determining factors, such as high insulin levels, alterations in lipid profile, and microalbuminuria, which are known to be prevalent in salt-sensitive hypertension, may explain, at least in part, the increase in cardiovascular risk observed in salt-sensitive patients.

Recent studies have highlighted the genetic and metabolic background of salt-sensitivity, a phenomenon with remarkable variability among human subjects [33], as well as among animal models [42]. A number of genetic, hormonal, and neuro-endocrine factors are involved in the salt-sensitivity of BP [43]. The sympathetic nervous system, the renin-angiotensin-aldosterone system, natriuretic peptides, insulin, leptin, and several endothelial mediators with endocrine activity may modify BP response to salt [15]. BP salt-sensitivity may be genetically inherited, as in some rare monogenic forms of hypertension, or influenced by several genetic polymorphisms involving sodium reabsorption in the nephron or acquired by the individual subjects in their lifetime. Aging amplifies the hypertensive effects of increased sodium intake [44]. The reason of this phenomenon may be the decrease in the kidney ability of concentrating sodium in the urine with increasing age, likely due to a decline in glomerular mass with age. Similarly, chronic kidney disease leads to an impairment of volume excretion and urine sodium-concentrating ability, thus enhancing the salt-sensitivity in its more severe forms. Individuals from African descent are at an increased risk for hypertension, despite plasma volume and cardiac index similar to white population [45]. The ability of concentrating sodium in urine after salt loading seems impaired in the blacks compared to whites [46], thus supporting salt-sensitivity as a more common cause of hypertension among blacks. BP salt-sensitivity also showed a positive association with obesity, being found higher among obese rather than in lean adolescents, and is reversed after weight loss [47]. Abdominal adiposity and the metabolic syndrome [48] are associated with an increased rate of sodium reabsorption by the kidney, an effect that is at least partially mediated by insulin and leptin [49].

Although salt-sensitivity of BP is a well-established phenomenon and the correlations of this phenotype with clinical features have been established, the pathophysiologic mechanisms leading to increased BP values have been long-time debated and have not yet been completely elucidated. Until recently, according to the classic concept of Guyton [50], the prevailing theory is that high salt intake leads to an expansion in circulating volumes, an increased cardiac output, and a rise in kidney perfusion pressure. The “pressure-natriuresis” mechanism tends to increase sodium output to restore the increased circulating volume to normality. Salt-sensitivity is thus conceived and explained by a relative ‘natriuretic handicap’ from the kidney, which is unable to produce a sufficient excretion of sodium to preserve sodium balance and volumes without sufficiently higher pressure. Hypertension may develop only when the excretory ability of the kidney is impaired and the relation between sodium excretion and BP is shifted toward higher values. Research into the possible physiological mechanisms determining salt-sensitivity has thus been driven mostly by a conceptual framework derived from the work of Guyton, which is also highlighted in a recent scientific statement from the American Heart Association on this topic [15].

The traditional view of sodium handling has been challenged by the finding of non-osmolar storage of sodium. The traditional framework assumes that sodium and chloride are osmotically active and cause water retention in amounts which preserve an unchanged osmolarity. A high-sodium diet may expand the extracellular volume until a steady state is reached, where sodium intake and output are balanced, with a significant increase in the quantity of total body water. However, rigorous studies have demonstrated that sodium may accumulate in the body without a concomitant retention of water, both in humans [51] and in experimental models [52]. Recent clinical research has highlighted that salt-sensitive and salt-resistant patients do not show any difference in circulating volumes, cardiac output, or sodium balance after salt loading [53]. This may be explained by non-osmolar storage of sodium, without considering the effect on water retention.

An alternative to the Guyton framework of the “pressure-natriuresis” and the “natriuretic handicap” was also developed [14]. Evidence has accumulated showing that impairment in vascular function may play a relevant role in salt-sensitive hypertension. In fact, abnormal responses of vascular resistance in the renal circulation are present after salt intake in salt-sensitive individuals, without an increase in sodium retention or cardiac output [46,54]. After acute or chronic increases in salt intake, salt-sensitive patients are not subject to an increase in sodium storage and do not increase the cardiac output, when compared to normal controls [55]. Then, the increase in BP induced by salt may be mediated by abnormalities in the vascular response to salt, in particular in peripheral and renal resistances, along with changes in sodium balance and in cardiac output [56]. In normal conditions, salt-resistant individuals may show a robust decrease in systemic vascular resistances after an increase in salt intake [57]. Specifically, in salt-sensitive individuals, the effect of salt includes a failure to induce a normal decrease in vascular resistances, which may show unchanged or even increased levels. Conversely, normal salt-resistant individuals are able to induce a vasodilatory response after an increase in salt intake [58], and then BP is maintained within normal values. Thus, the preservation of normal BP levels under salt loading appears to be independent from the ability of salt resistant subjects to rapidly excrete a salt load or to better manage better the balance of sodium, of circulating volume, and of cardiac output than salt-sensitive individuals, as hypothesized in a classical view of salt-sensitivity. According to these recent theories, these alternative physiological mechanisms involved in the phenomenon of salt-sensitivity [14] could be framed in a large unifying view of the salt-sensitivity phenomenon and explain the recent observations on the effects induced by salt in the arterial vessel wall.

## 4. Sodium Intake and Sympathetic Activity

Diet with high salt supply can modulate the activity of the autonomic nervous system, especially sympathetic activity, in several ways. A previous study of our research group [35] showed, in salt-sensitive hypertensive patients, different changes in autonomic cardiovascular control at different levels of sodium loading. Salt-Sensitivity Index (SSI) was calculated in 34 essential hypertensive patients [35]. SSI is the ratio of the change in brachial mean arterial pressure (∆MAP), between the high- and the low-sodium diet periods, with the corresponding change in urinary sodium excretion rate (∆UNaV, expressed in mmol/L/day), multiplied by a factor of 1000. Autonomic cardiovascular control was evaluated by spectral analysis of beat-by-beat finger BP and pulse interval variability, and by the related assessment of spontaneous baroreflex sensitivity (sequence technique) [59,60,61]. The results of these studies indicate a better parasympathetic cardiac modulation (quantified by baroreflex sensitivity and indexes of heart rate variability in the high frequency band) associated with lower SSI. These results underline the presence of greater sympathetic activation in salt-sensitive patients; in fact, they exhibit the physiological reciprocal behavior that usually characterizes sympathetic and sympathetic cardiac modulation. [62]. In fact, a sodium-rich diet is reported in subjects with lower SSI who are also characterized by a preserved autonomic cardiovascular modulation. Therefore, an increased dietary salt supply can induce a reflex reduction of sympathetic efferent activity if reflex cardiovascular regulation is physiologically preserved, activating cardiopulmonary receptors through an increase in plasma volume [63]. An opposite condition occurs in the presence of low sodium intake [24]. However, this neural regulation has not been described in patients with the highest degree of salt-sensitivity. No changes in their impaired autonomic cardiovascular control are associated with changes in sodium intake. In conclusion, the increased BP associated with excessive sodium intake observed in hypertensive patients, characterized by high salt-sensitivity, may be due to the impairment of their baroreflex function or to their inability to increase baroreflex sensitivity and reduce sympathetic activity in response to the increase in plasma volume, determined by sodium loading [24,62,63,64,65,66,67,68]. More recently, we have also reported a blunted vagal control of heart rate in young normotensive individuals with a higher degree of sodium-sensitivity, when facing a high-salt diet [36].

## 5. Salt-Induced Vasodysfunction

In the pathophysiological explanation of the salt-sensitivity of BP a dysfunction in vascular modulation has also been hypothesized. An increased salt intake may clearly provoke an expansion in circulating volumes, an increase in flow and BP values, and thus an adverse remodeling of arterial wall mediated by the mechanic load through shear stress and an increase in wall tension. Beyond that, several experimental and clinical studies have recently demonstrated the adverse effects of high sodium intake in the microvascular circulation [13].

In experimental animal studies, salt intake was associated with microvascular rarefaction in normotensive and hypertensive rats, resulting from structural alterations and differing from the degenerative processes observed in experimental animals with chronic hypertension that are characterized by microvascular rarefaction [69]. Moreover, apart from microvessel rarefaction, a reduced arterial vasodilator capacity developed with high-salt diet has also been described in rats with hypertension induced by a reduced renal mass, which and was restored with low-salt diet [70]. Additional vascular effects of a high sodium intake include the potentiation of local vasoconstrictive effectors, such as alterations of endothelial Ca2+ signaling [71] or an abnormal high production of 20-hydroxyeicosatetraenoic acid [72]. The upregulation induced by a high salt intake of the cytochrome P450 ω-hydroxylase 4A)/20-hydroxyeicosatetraenoic acid system results in elevated oxidative stress and a reduced nitric oxide bioavailability, causing vascular dysfunction [73], and may thus be a key mediator linking increased salt intake to microvascular dysfunction.

A series of human studies have identified alterations in the small arteries and endothelial function in relation to salt intake. Impaired vasodilatation of the small vessels has been shown to occur in conditions of high salt intake [74]. In young, healthy normotensives, salt loading impaired vascular endothelial function along with left ventricular mechanical relaxation [75]. These results were confirmed in normotensive adults, where sodium-induced impairments in microvascular function were observed. The microvascular function was improved by the administration of the anti-oxidant ascorbic acid, suggesting a role of oxidative stress in this process [76]. An impairment in endothelial function was observed in the brachial arteries of healthy volunteers subjected to high-salt diet, with a switch in the mediator of vasodilation in the microcirculation to a non-nitric oxide-dependent mechanism, which was restored with acute exercise [77]. Moreover, in young normotensives, intravenous sodium loading had direct adverse effects on the endothelial surface layer by increasing microvascular permeability to albumin, independently from BP [78]. Furthermore, an imbalance between cardiac output and vascular resistances in salt-sensitive subjects, determined by the failure to adequately lower vascular resistances after increase of sodium intake, has been described in young normotensive subjects [36], thereby confirming data collected in normotensive black individuals [79].The restriction in dietary sodium in middle aged hypertensives largely reversed microvascular endothelial dysfunction by increasing nitric oxide and tetrahydrobiopterin bioavailability, and by the reduction of oxidative stress, supporting the role of a vascular protection induced by salt restriction beyond that attributable to its BP-lowering effects [80].

A link was hypothesized between the described vascular impairments and abnormalities in interstitial sodium storage [53]. The emerging magnetic resonance imaging–based techniques that directly detect Na^+^ in tissues [81] have recently provided confirmation and further evidence about the compartmentalized sodium storage in humans, also in relation to cardiovascular morbidities [82]. Recent experiments [83,84,85] have shown that sodium homeostasis is regulated by negatively-charged glycosaminoglycans in the skin interstitium, where sodium is bound to glycosaminoglycans without causing an effect on extracellular volume. Negatively-charged glycosaminoglycans in the skin may be important for non-osmotic sodium accumulation and explain the observation a positive sodium balance without a concomitant volume expansion [86]. The skin is thus known to represent the main site for the storing of sodium in the body, with a buffering capacity adapting to changes in salt intake in which skin glycosaminalgycans have been shown to have the most relevant role [83]. The relationship between skin deposition of sodium and hypertension may be mediated by a molecular mechanism in which vascular-endothelial growth factor-C (VEGF-C) is the most relevant mediator. The hypertonicity of skin interstitial space, which develops during high salt intake, is accompanied by newly-developed lymphatic vessels and an increased density and hyperplasia of the lymphocapillary network, a process regulated by macrophages releasing the osmosensitive transcription factor, that in turn induces the release the VEGF-C [87,88], which enhances the production of endothelial nitric oxide synthase and of nitric oxide. Failure of this regulatory mechanism, which enhances sodium excretion via lymphatics and regulates vascular tone by increasing endothelial nitric oxide synthase protein expression, may lead to a salt-sensitive BP response [89].

Although the molecular pathway involving VEGF-C has been the most studied in animal models to explain the link between skin sodium and hypertension, other mechanisms have been proposed to play a role in this relationship. The increase in salt intake has been shown to induce the rarefaction of the skin microcapillary network in different racial groups [90], and to increase the reactivity of skin vessels in response to angiotensin-2 and noradrenaline [91]. Other works suggest that the hypoxia inducible factor (HIF) may represent a key regulator of vascular tone of the skin [83], although its role has been mainly studied in the renal medulla until now [92]. Although further works are needed to clarify the underlying mechanisms involved in this relationship, the role of skin in regulating BP, and by mediating a vasodilatory response.

## 6. Sodium Intake and Arterial Stiffness

The close relationship between high dietary salt content, arterial hypertension, and increased stiffness of the large arteries is not a recent discovery. In fact, Huang Ti Nei Ching Su Wein, a wise Chinese doctor who lived 3700 years ago, already argued in his studies: *“…therefore if large amounts of salt are taken, the pulse will stiffen and harden”*. He was indeed right. In fact, in the following centuries it was confirmed that a high plasma serum sodium deeply affects the functional peculiarities of the large elastic arteries [93], and is associated with a relative increase in systemic peripheral resistance. Additionally, an effect of sodium on small resistance arteries has also been demonstrated [94]. Among the various studies that have investigated this topic, we recall the Avolio et al. research performed in the 1980s [95,96]. In their papers, urinary sodium excretion was used as a surrogate for daily dietary intake. Comparing rural and urban populations, an average excretion of 13.3 g/24 h of NaCl was found in the cities, and 7.3 g/24 h in the rural population. This difference reflects different dietary habits in the two recruited groups, which are physiologically expressed in significantly lower carotid-femoral pulse (PWV) wave velocity in the rural community [95]. Hypertension also had a higher incidence in the urban group. This topic has been further investigated in a following study on normotensive subjects [96]. Even in this case, lower aortic PWV values were detected in subjects who followed a low-salt diet compared to a reference group. As in the previous study, the difference between groups in PWV was not dependent on BP levels. In a number of studies involving hypertensive patients [97,98,99,100], aortic PWV values were significantly lower in the low-salt group than in the high-salt group; however, other studies in which no significant difference in PWV has been described in relation to dietary sodium intake can be found in the literature [101,102,103,104,105,106,107]. In almost all of these latter randomized controlled trials, however, the relatively small number of enrolled patients and the relatively short duration of a given level of sodium intake with the diet assigned to each group were probably the main factors affecting the failure in reaching a statistically significant difference in PWV between high- and low-salt groups. A meta-analysis recently published by D’Elia et al. tried to better clarify the relationship between sodium intake with diet and arterial stiffness [108]. The results of this study show that an average reduction in salt intake of 5 g/day is associated with a 2.8% reduction in carotid-femoral PWV. The authors also showed how this PWV reduction was independent of the reduction in BP values in hypertensive and/or pre-hypertensive middle-aged subjects. Since the relationship between arterial stiffness and sodium intake has been mostly evaluated during salt-intake manipulations, data on the effects of sodium-sensitivity condition on PWV are scarce.

**Figure 1:**
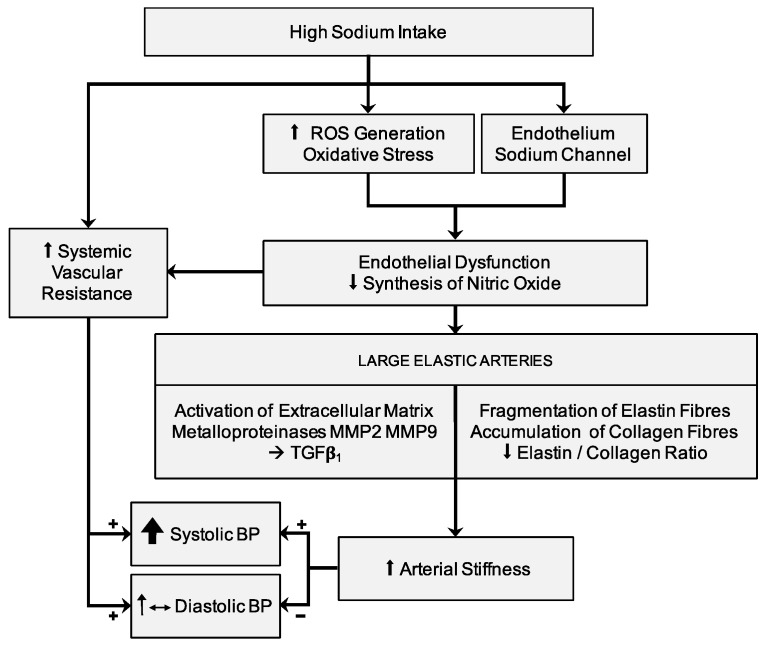
Relationship between high salt intake with diet, blood pressure, and arterial stiffness. Abbreviations: BP, blood pressure; MMP, matrix metalloproteinases; ROS, reactive oxygen species; TGF, transforming growth factor.

The relationship between high BP values and arterial stiffness is described and confirmed in several studies [109,110]. High BP values that persist for a prolonged time interval lead to progressive structural changes in the arterial wall of the large elastic arteries, with consequent increase in arterial stiffness. In particular, the increase in the expression of collagen fibers, and the consequent reduction in the ratio between elastin and collagen fibers, can cause the progressive increase in arterial wall stiffness. In this context, the aforementioned alterations in arterial stiffness —independent of arterial pressure and due to a sodium-rich diet for prolonged periods of time—are associated with the pathophysiologically-expected interrelations between BP and PWV. It seems really difficult to discriminate between BP-dependent and BP-independent variations of the viscoelastic properties of large arteries in relation to the effects of sodium intake. An excessive sodium intake with diet induces alterations in the extracellular matrix of arterial wall, favoring a process of arterial stiffening (Figure 1). Endothelial dysfunction [111,112] and oxidative stress [113] related to high sodium intake can cause vascular damage through a pressure-independent mechanism [112]. The mechanical properties of the aorta and large elastic arteries depend on the relationship between the principal components of the extracellular matrix in arterial wall: i.e. the elastin and collagen fibers. Thus, the elastin and collagen fibers ratio characterizes the viscoelastic properties of large arteries and is regulated by matrix metalloproteinases (MMPs) [114]. A high sodium intake causes an activation of extracellular matrix metalloproteinases MMP2 and MMP9, leading to stimulation of TGFß-1 [112,114,115], inducing thinning and breakage of elastin fibers and a decrease in the elastin and collagen ratio. On the other hand, the overexpression of TGFß-1 inhibits collagenase production [116] and develops a fibrogenic effect on the extracellular matrix in the arterial wall, altering its mechanical properties [117]. An important role in the expression of the viscoelastic properties of large arteries seems to be linked to the balance between MMP2 and MMP9 (both favoring the accumulation of collagen) [114,115,118] with MMP8 and MMP13 (which instead promote collagen degradation) [119,120]. Accelerated arterial fibrosis may be responsible for increased arterial stiffness and for amplification of aging-related vascular damage.

The renin-angiotensin-aldosterone system (RAAS) plays also a major role in the regulation of the mechanical properties of large elastic arteries, activating the MMPs [121] and increasing collagen I synthesis [122]. High sodium intake with diet also seems to be able to stimulate aortic Angiotensin II receptor type 1 (AT_1_-receptors) [123], and the vascular damage induced by excessive sodium intake can be modulated by genetic factors, in particular by the polymorphism of AT_1_-receptor genes [124] and aldosterone synthase genes [125]. These genetic polymorphisms appear to be of particular relevance in the elderly and in the hypertensive patients [117,124]. The highest mortality associated with excessive dietary sodium intake was significantly reduced in rodents when the high-sodium diet was associated with the intake of selective angiotensin II blockers [126,127].

## 7. Conclusions

The worldwide usual sodium intake ranges between 3.5–5.5 g per day (corresponding to 9−12 g of salt per day), with marked differences at a global level. Recommendations were made by the World Health Organization to limit sodium intake to approximately 2.0 g per day (equivalent to approximately 5.0 g salt per day) in the general population [5], and a particular effort in reducing salt intake should be made in the hypertensive population, which counts more than a billion patients globally. A reduction in salt intake can have a favorable effect on the cardiovascular system, inducing a reduction in BP values in hypertensive patients, but also with possible benefits in the vascular function and in the viscoelastic properties of the large arteries. Adequate attention should be paid to arterial structure and function when evaluating the cardiovascular outcomes of salt intake and of programs for salt reduction in the diet.
